# smoothHR: An R Package for Pointwise Nonparametric Estimation of Hazard Ratio Curves of Continuous Predictors

**DOI:** 10.1155/2013/745742

**Published:** 2013-12-12

**Authors:** Luís Meira-Machado, Carmen Cadarso-Suárez, Francisco Gude, Artur Araújo

**Affiliations:** ^1^Center of Mathematics, University of Minho, Campus de Azurem, 4800-058 Guimarães, Portugal; ^2^Department of Statistics and O.R., University of Santiago de Compostela, 15782 Santiago de Compostela, Spain; ^3^Clinical Epidemiology Unit, University Clinical Hospital of Santiago de Compostela, 15782 Santiago de Compostela, Spain

## Abstract

The Cox proportional hazards regression model has become the traditional choice for modeling survival data in medical studies. To introduce flexibility into the Cox model, several smoothing methods may be applied, and approaches based on splines are the most frequently considered in this context. To better understand the effects that each continuous covariate has on the outcome, results can be expressed in terms of splines-based hazard ratio (HR) curves, taking a specific covariate value as reference. Despite the potential advantages of using spline smoothing methods in survival analysis, there is currently no analytical method in the **R** software to choose the optimal degrees of freedom in multivariable Cox models (with two or more nonlinear covariate effects). This paper describes an **R** package, called smoothHR, that allows the computation of pointwise estimates of the HRs—and their corresponding confidence limits—of continuous predictors introduced nonlinearly. In addition the package provides functions for choosing automatically the degrees of freedom in multivariable Cox models. The package is available from the **R** homepage. We illustrate the use of the key functions of the smoothHR package using data from a study on breast cancer and data on acute coronary syndrome, from Galicia, Spain.

## 1. Introduction

An important aim in longitudinal medical studies is to study the possible effect of a set of prognostic factors on the course of a disease. In many of these studies, some of the prognostic factors may be continuous and their effects can be unknown. A classical approach for studying these effects is through the Cox regression model (Cox [1], Kalbfleisch and Prentice [2]). One possible approach allowing for nonlinear effects in the Cox model is to express the hazard as an additive Cox model (see, e.g., Hastie and Tibshirani [3], Gray [4], Huang et al. [5], and Huang and Liu [6]). In this paper, we use natural cubic regression splines (de Boor [7]) and penalized splines (P-splines, Eilers, and Marx [8]) to reflect the nature of continuous covariate effects in the additive Cox model. One of the most commonly used measures of this effect is the hazard ratio (HR) function. Cadarso-Suárez et al. [9] proposed a flexible method for constructing smoothing hazard ratio curves with confidence limits, which facilitates the expression of the results in a manner that is standard in clinical survival studies. The authors suggest the use of an additive Cox model where the effects of continuous predictors on log hazards are modeled nonlinearly using P-splines. This paper describes the R-based smoothHR (available from the Comprehensive R Archive Network at http://CRAN.R-project.org/package=smoothHR) package's capabilities for implementing pointwise estimates of HRs as well as their corresponding confidence limits. Numerical and graphical output can easily be obtained. The main feature of the package is its use for continuous predictors introduced nonlinearly in an additive Cox model but it can also be used when the predictor is introduced with a linear effect.

One disadvantage of natural cubic splines or penalized splines for modelling a continuous covariate's effect is the difficulty in choosing the number and location of the knots between which the smooth line is drawn. An arbitrary choice of number of knots and/or arbitrary knot location can mask important features in the data. While too many knots can lead to oversmoothing, too few can lead to undersmoothing. Within the **R**-function *pspline* (available in the survival package) two automatic selection criteria for selecting the optimal degree of smoothing (or equivalently, the optimal degrees of freedom) with P-splines are implemented: one is based on minimizing Akaike's Information Criterion (AIC, Akaike [10]) and the other one is based on minimizing a corrected version of this (AICc, Hurvich et al. [11]). Minimization of these two criteria can easily be achieved in the univariate setting but becomes increasingly complex in the multivariable setting. The Bayesian Information Criterion (BIC) proposed by Schwarz [12], and extended by Volinsky and Raftery [13] to survival data, can also be used in this context. In this paper we propose a function called *dfmacox*, within the package smoothHR, that provides the optimal number of degrees of freedom in the multivariable Cox model. The optimal degree of smoothing is obtained by minimizing any of the following criteria: AIC, AICc, or BIC. This function can also be used for natural cubic regression splines.

The rest of the paper is organized as follows.  Section 2 introduces the Cox proportional hazards model.  Section 3 describes the procedure to obtain the confidence limits for the hazard ratio curve taking a specific covariate value as reference. An overview of the features and functions of the package smoothHR is given in Section 4. Illustrative real data application is provided in Section 5 using two databases from Galicia, Spain. The main body of the paper ends with a discussion section.

## 2. The Additive Cox Model

The effect of prognostic factors in survival analysis is generally modeled using the Cox proportional hazards model. Formally, the Cox model assumes that the hazard function can be written as

(1) $$\begin{array}{c} \alpha \left(t;Z\right)=\alpha_{0}\left(t\right)\exp\left(\beta^{T}Z\right), \end{array}$$

where *t* is time, *Z* = (*Z*_1_,…, *Z*_*p*_)^*T*^ is a *p*-dimensional vector of time-fixed covariates, *β* is the associated vector of unknown regression parameters, and *α*_0_(*t*) a nonnegative baseline hazard function.

The effect of covariates estimated by any proportional hazards model can thus be reported as hazard ratios (HRs). The adjusted HR for a subject with (continuous) covariate value *Z*_*i*_ compared to a subject with covariate value *z*_*i*,ref_ is given by

(2) $$\begin{array}{c} \text{H}{\text{R}}_{i}\left(Z_{i},z_{i,\text{ref}}\right)=\exp\left(\beta_{i}\left(Z_{i}-z_{i,\text{ref}}\right)\right). \end{array}$$

The logarithm of the hazard ratio curve is then reduced to a straight line, indicating that the expected change in risk for a (*Z*_*i*_ − *z*_*i*,ref_) change in *Z*_*i*_ is a constant value (the well-known proportional hazards assumption).

For the Cox model (1) the effect of prognostic factors is assumed to have a log-linear functional form. However, the incorrect functional form for a covariate can lead to a diagnosis of nonproportional hazards (Therneau and Grambsch [14]) or to erroneous statistical conclusions (bias and decreased power of tests for statistical significance) (Struthers and Kalbfleisch [15], and Anderson and Fleming [16]). The need to overcome these problems has led to many developments (Hastie and Tibshirani [3], Kneib and Fahrmeir [17], Huang et al. [5], and Huang and Liu [6]). One possible approach to incorporate nonlinear effects into the Cox model is to express the log hazard as an additive function:

(3) $$\begin{array}{c} \alpha \left(t;Z\right)=\alpha_{0}\left(t\right)\exp\left(\sum_{i=1}^{q}f_{i}\left(Z_{i}\right)+\sum_{i=q+1}^{p}\beta_{i}Z_{i}\right), \end{array}$$

where the first *q* covariates are continuous and introduced nonlinearly through (unknown) smooth functions, *f*_*i*_, and the remaining ones are covariates introduced parametrically in the model. The major feature making model (3) more suitable in most applications is that it allows for nonlinear, smooth effects for continuous predictors, leading to a considerable amount of additional flexibility. The effects can efficiently be modeled using natural regression splines (de Boor [7]) or penalized splines (P-splines; Eilers and Marx [8]). The general idea is to approximate the functions by linear combinations of B-spline basis functions (de Boor [7]).

### 2.1. Smoothers and Controlling the Amount of Smoothing

Natural cubic splines have proven to be a good choice, leading to twice continuous differentiable (smooth) functions. These functions impose monotonicity in the tail regions (with constraints to be linear beyond certain extreme observations), whereas in regions where data are dense, monotonicity is effectively imposed by the data themselves. The curve complexity is governed by the number of degrees of freedom (one fewer than the number of knots), which is given by the number of basis function, equal to the number of fitted regression coefficients. Interpretation of the regression coefficients is not particularly interesting in itself but their estimates allow for visualizing the spline fit. The ns function in package splines accomplishes the fit of model (3) using natural splines. One important issue is the choice of the number of degrees of freedom and the placement of the knots. The Akaike Information Criterion (AIC) proposed by Akaike [10] and the Bayesian Information Criterion (BIC) proposed by Schwarz [12] are two of the more popular criteria for choosing a best model for a given data set. These two criteria are based on the log likelihood but can be extended to handle the Cox proportional hazards model by using the log-partial likelihood. The choice of the degrees of freedom in additive Cox models can be accomplished by comparing models with different degrees of freedom and choosing the model with minimum AIC or BIC scores. In the Cox proportional hazards model, AIC and BIC scores are calculated as

(4) $$\begin{array}{c} \text{AIC}=-2\times \text{LPL}+2\times \text{df},\\ \text{BIC}=-2\times \text{LPL}+\log\left(n\right)\times \text{df}, \end{array}$$

where LPL is the maximum log-partial likelihood of the fitted model, df represents the equivalent degrees of freedom of the fit, and *n* is the number of observations in a given data set. Since there are censored observations in survival data, Volinsky and Raftery [13] modified/corrected the penalty coefficient log⁡(*n*) in BIC defined for censored survival models. They used the number of uncensored observations in the penalty term instead of the sample size *n*. Throughout this paper we will make use of the corrected version of Bayesian Information Criterion (BIC) proposed by Volinsky and Raftery [13]. The AIC and BIC scores have similar forms, differing only in the penalty coefficient. In both scores, the first term rewards goodness of fit whereas the second is a penalty that is an increasing function of the number of estimated parameters (df). The penalties in the expressions of the AIC and BIC (2 × df and log⁡(*n*) × df, resp.) discourage overfitting. Methods for knot placement have been developed in the literature (Durrleman and Simon [18], Friedman [19], and Royston and Parmar [20]). The ns function uses default knot positions which are placed at predefined percentiles of the survival times.

There have been doubts about the penalty in the Akaike Information Criterion (AIC). Too many degrees of freedom will lead to extra variability whereas too few degrees of freedom could mean serious modeling bias and missing important explanatory features in the analysis. Thus selecting “optimal” number of degrees of freedom is a statistical balancing act between bias and variance. Usually, the results point to the fact that AIC score leads to overfitting choosing models with larger number of degrees of freedom, regardless of *n*. Because of this, this criterion may not be suitable if the number of data points is small; then some correction is often necessary. Hurvich et al. [11] show that in nonparametric regression, the AIC can underpenalize, leading to models with very large number of degrees of freedom, especially when data are dispersed. They suggest a corrected AIC (AICc) which uses *n* × (df + 1)/(*n* − (df + 2)) as the correction term instead of df:

(5) $$\begin{array}{c} \text{AICc}=-2\times \text{LPL}+\frac{\left(2\times n\times \left(\text{df}+1\right)\right)}{n-\left(\text{df}+2\right)}. \end{array}$$

In the case of a Cox model, *n* is replaced by the total number of events (Therneau and Grambsch [14]).

Penalized spline methods have gained recent popularity due to Eilers and Marx [8] when they introduce penalties to the B-splines. The idea is to represent the curves *f*_*i*_ in model (3) by an overfitted spline function and to control the smoothness by imposing a penalty term to the model's likelihood function. Penalized spline models are a popular statistical tool in Cox additive regression models due to their flexibility and computational efficiency. The **R**-function *pspline* in package survival can be used to fit model (3). One particular concern in fitting P-splines is the selection of reasonable values for the smoothing parameters. By default, in the **R**-function *pspline* implementation, the amount of smoothing for a continuous covariate effect is given by a total of four degrees of freedom. The AIC criterion and the corrected AIC option are also available in the **R**-function *pspline*. Minimization of the criteria AIC and AICc can easily be achieved in the univariate setting but becomes increasingly complex in the multivariable setting.

### 2.2. Obtaining the Optimal Degrees of Freedom

We propose the following procedure to obtain the (multivariable) degrees of freedom that minimize each one of the three criteria mentioned in Section 2.1. This procedure consists of the following steps.

**Step 1**

Set the maximum value for the degrees of freedom for each (continuous) covariate. This value can be fixed by the user. By default, when penalized spline is used, the corrected AIC from Hurvich et al. [11] obtained in the corresponding univariate additive Cox model is used.

**Step 2**

Set the minimum value for the degrees of freedom. This value is set to 1 if the selected smoother is “natural splines” (since the degrees of freedom must be integer) and strictly greater than 1 for “penalized splines.”

**Step 3**

For each covariate a vector with three values for the degrees of freedom is created: (minimum, mean = (minimum + maximum)/2, maximum). Then, a data frame is created from all combinations of the supplied vectors (one for each covariate), the corresponding Cox model is fitted, and the score for the corresponding criterion is obtained.

**Step 4**

For each covariate a vector with two values for the degrees of freedom is created based on the results (scores) obtained in Step 3 (minimum and mean, or mean and maximum).

**Step 5**

Repeat Step 3 and Step 4 a number of times, *n*  times. By default *n*times = 5. The degrees of freedom are obtained from the model minimizing the selected criterion.

If natural splines are used, and the minimum value equals 1 (Step 1) and the maximum equals 20 (Step 2), then *n*times = 4 ensures that the “true” minimum of the (AIC, AICc, or BIC) criterion will be achieved. Such target (the “true” minimum of the criterion) will be unknown for penalized splines (because the degrees of freedom are nonnegative real numbers). However, our procedure guarantees that the obtained degrees of freedom will be close to that target.

This methodology is implemented in the **R**-function called *dfmacox*, within the package smoothHR. This approach is based on minimization of the three criteria (AIC, AICc, and BIC) and can be used with penalized splines or natural splines. The P-spline approach is computationally intensive for modeling the unknown functions *f*_*i*_ in (3), especially if the number of smooth terms *q* in the model is large.

In general, the AIC has a big chance of choosing too much degrees of freedom. Because of this, the corrected AIC may be preferable. On the other hand, for small samples, the BIC often chooses models that are too simple (i.e., with few degrees of freedom). Thus, it might be better to use AICc and BIC together for selecting the degrees of freedom. It is worth mention that some authors (Cadarso-Suárez et al. [9] and Govindarajulu et al. [21]) suggest a compromise between the AIC criterion and prior ideas of biologic plausibility, which would support a more monotonic (i.e., less degrees of freedom) curve.

An alternative approach that allows for determining the smoothing parameters has been proposed by Kneib and Fahrmeir [17]. Such approach is based on a mixed model representation of penalized splines. The basic idea is to interpret the penalty term as a random effects distribution assigned to the vector of regression effects, which effectively turns the smoothing parameter into a variance component. Concepts from mixed model methodology such as restricted maximum likelihood (REML) estimation can then be adapted to the additive hazard model setting. Cross-validation (CV) or generalized cross-validation (GCV) can serve as alternative approaches (Wood [22], and Tsujitani and Tanaka [23]).

## 3. Smooth Hazard Ratio Curves

The HR curve for a continuous predictor *Z*_*i*_ in an additive Cox model (3) can be written as

(6) $$\begin{array}{c} \text{HR}\left(Z_{i},z_{i,\text{ref}}\right)=\exp\left(f_{i}\left(Z_{i}\right)-f_{i}\left(z_{i,\text{ref}}\right)\right), \end{array}$$

where *z*_*i*,ref_ is a specific value of the predictor taken as the reference. A natural estimate of the adjusted HR curve HR(*Z*_*i*_, *z*_*i*,ref_) in (6) can be constructed as ${\hat{\text{HR}}}_{i}(Z_{i},z_{i,\text{ref}})=\exp({\hat{f}}_{i}(Z_{i})-{\hat{f}}_{i}(z_{i,\text{ref}}))$ by replacing *f*_*i*_(·) by the corresponding P-spline estimate, ${\hat{f}}_{i}(\cdot )$ (or any other smoother). After taking logarithms for simplicity, the asymptotic variance of $\text{Ln}\hat{\text{HR}}(Z_{i},z_{i,\text{ref}})$ can be expressed in terms of the covariance matrix of the P-spline estimate ${\hat{f}}_{i}(\cdot )$:

(7) Var⁡(LnHR^(Zi,zi,ref))=Var⁡(f^i(Zi))+Var⁡(f^i(zi,ref))−2Cov(f^i(Zi),f^i(zi,ref)),

where the asymptotic covariance matrix takes the form of *H* ^−1^ *IH* ^−1^, with *I* being the usual observed information and *H* = *I* + *P*, where *P* is the second derivative matrix of the penalty function (see details in Eilers and Marx [8], Gray [4], or Cadarso-Suárez et al. [9]). Finally, assuming normality, (1 − *α*)100% pointwise confidence limits can be constructed around the HR_*i*_(*Z*_*i*_, *z*_*i*,ref_) curve

(8) $$\begin{array}{c} \exp\left(\text{Ln}\hat{\text{HR}}\left(Z_{i},{\text{z}}_{i,\text{ref}}\right)\right)\pm z_{1-\alpha /2}\text{SE}\left(\text{Ln}\hat{\text{HR}}\left(Z_{i},z_{i,\text{ref}}\right)\right), \end{array}$$

where $\text{SE}(\text{Ln}\hat{\text{HR}}(Z_{i},z_{i,\text{ref}})=\sqrt{\left(\mathrm{Var}(\text{Ln}\hat{\text{HR}}(Z_{i},z_{i,\text{ref}}))\right)}$ is the standard error of $\text{Ln}\hat{\text{HR}}\;(Z_{i},z_{i,\text{ref}})$ and *z*_1−*α*/2_ is the upper quantile of the standard normal distribution.

We developed an **R** package, called smoothHR, for computing the HR curve and to provide “optimal” degrees of freedom in multivariable additive Cox models. Detailed information about this software is shown in the next section.

## 4. Software Description

The **R**-based package smoothHR contains functions that provide pointwise estimates of Cox model HR curve for continuous predictors as well as the corresponding confidence limits. Though penalized spline smoothing is suggested the software is able to deal with other smoothers such as natural splines or B-splines (within the **R**-functions bs and ns available in the package spline). For the moment, function *dfmacox* only deals with natural splines (ns) and P-splines (*pspline*).

This package is intended to be used with the **R** statistical program (R Development Core Team [24]). Our package is composed of five functions that allow users to obtain both numerical and graphical outputs.  Table 1 provides a summary of the functions in this package. Details on the usage of these functions can be obtained within the corresponding help pages.

As mentioned in Section 2, controlling the amount of smoothing is not a problem in univariate additive Cox models. In the multivariable case, however, various problems may arise. In this paper, we propose a new function called *dfmacox*:

 
dfmacox (time, time2 = NULL, status,
   
 nl.predictors, other.predictors,
   
smoother, method, mindf = NULL,
   
maxdf = NULL, ntimes = NULL, data)

This function provides an approach to obtain the degrees of freedom for multivariable additive Cox models. The continuous predictors to be introduced nonlinearly must be included in the argument nl.predictors (as a vector), whereas the remaining predictors (continuous or not) are included in argument other.predictors. This function returns a list with the degrees of freedom for the spline smoothing (cubic natural splines, if smoother="ns", and penalized splines, if smoother="pspline") and the corresponding fit of the Cox model (3). The degrees of freedom are obtained by minimization of the following criteria: (a) the AIC criterion (if method="AIC"), (b) based on the adaptation of the corrected AIC proposed by Hurvich et al. [11] (if method="AICc"), and (c) the BIC criterion (if method="BIC").

The main function of the package, smoothHR, can then be used. If the Cox model has been fitted, then the smoothHR function only needs arguments data and coxfit. Otherwise, arguments time and time2 (optional, for counting process data) and status are required:

 
smoothHR (data, time = NULL, time2 = NULL,
   
status = NULL, formula = NULL, coxfit,
   
status.event = NULL)

Afterwards, the plot function can be used to plot flexible hazard ratio curves allowing nonlinear relationships between continuous predictors and survival. Results are expressed in terms of hazard ratio curves, taking a specific covariate value as reference. Confidence limits for these curves are also derived:

 
plot (x, predictor, prob = NULL,
   
pred.value = NULL, conf.level = 0.95,
   
round.x = NULL, ref.label
   
= NULL, col, main, xlab, ylab, lty,
    
 xlim, ylim, xx,*⋯*)

The reference value can be specified using argument pred.value. Alternatively, the reference value can be defined automatically as the value at which the HR curve has a minimum (prob = 0) or a maximum (prob = 1).

Numerical output (including estimates for the hazard ratio and corresponding confidence limits) can be obtained within the *predict* function of the package. Finally, the *print* function gives details about the Cox model such as the fitted model and the proportional hazards assumption (Grambsch and Therneau [25]).

These functions will be illustrated in the next section using two real data sets.

## 5. Examples of Application

To illustrate our software, we use data from two databases from Galicia, Spain. In the first database we reanalyzed survival data from 811 patients admitted to the coronary care unit of the Santiago University Teaching Hospital between September 2003 and March 2007 with a diagnosis of acute coronary syndrome (ACS). In the paper by Cid-Alvarez et al. [26] the authors study the predictive capacities of admission and fasting glucose among patients with and without diabetes. A J-shaped dependence of the all-time mortality hazard ratio on fasting glucose was found among patients with no history of diabetes.

In addition to the acute coronary syndrome we consider data on 584 incident cases of breast cancer, diagnosed at the Santiago University Teaching Hospital (Cadarso-Suárez et al. [9]) from 1991 to 2000. In this data set Cadarso-Suárez et al. [9] have found DNA measurements of worth prognostic for predicting recurrence. In addition they found a significant nonlinear effect of this prognostic factor while using penalized smoothing splines in a Cox proportional hazards model. Plots were given for the smooth hazard ratio curve taking the value 1 as the reference. Below we will illustrate how such a plot can be obtained using our R package smoothHR.

### 5.1. Acute Coronary Syndrome Data

In the study by Cid-Alvarez et al. [26] the authors assess and compare the abilities of admission and fasting glucose to predict the death of ACS patients, distinguishing those with or those without a previous diagnosis of diabetes. In their analysis, the nonlinear relationships between glucose levels and risk of death were modeled by means of natural cubic spline Cox analyses.

**Figure 1:**
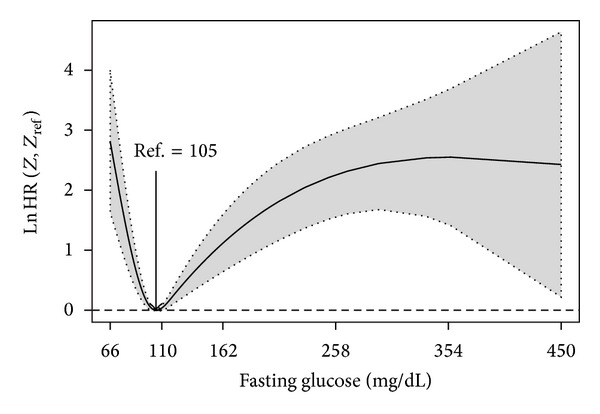
Nonparametric estimates of the dependence of all-time risk of death on fasting glucose among ACS patients without a prior diagnosis of diabetes mellitus (log hazard ratio, with 95% confidence limits, unadjusted analysis).

Below we present the input commands for obtaining the log hazard ratio curve of the dependence of all-time risk of death on fasting glucose among ACS patients without a prior diagnosis of diabetes mellitus. The corresponding plot is shown in Figure 1. It is clear from this figure that among the 558 patients with no history of diabetes there was a J-shaped dependence of the all-time mortality hazard ratio on fasting glucose: hazard was lowest at 105 mg/dL (5.8 mmol/L; to convert mg/dL of glucose to mmol/L, divide by 18.). Estimates for the log hazard ratio and the corresponding confidence limits were obtained using the *predict* function:

R> library ("survival")
R> library ("smoothHR")
R> heart2<-heart [heart$diabetes==0,]
R> df1<-dfmacox (time= "time", status
 
= "exitus", nl.predictors = c ("fasting"),
 
smoother = "ns",  method = "AIC",
 
 data = heart2)
R> df1$df
 
[1] 3
R> hr1<-smoothHR (time = "time", status
 
= "exitus", formula = ~ns (fasting, df =
 
 df1$df), data = heart2)
R> xx<-c (66, 110, 162, 258, 354, 450)
R> plot (hr1, predictor = "fasting",
 
prob = 0, conf.level = 0.95, ref.label =
 
"Ref.", xaxt = "n", main = "", xlab
 
= "Fasting glucose(mg/dL)")
R> axis (1, xx)

R> pdval<-c (70, 80, 90, 100, 110, 120,
 
140, 180, 250, 400)
R> predict (hr1, predictor = "fasting",
 
prob = 0, conf.level = 0.95,
 
prediction.values  = pdval,
 
ref.label = "Ref.")

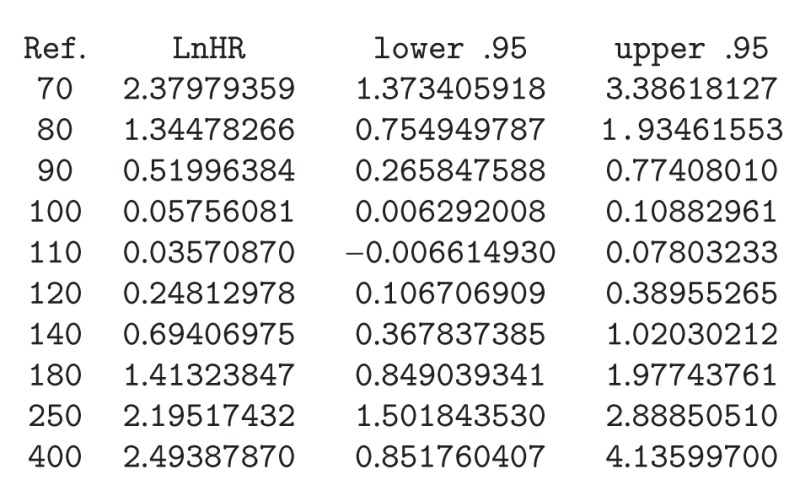


Various multivariable Cox models of all-time risk of death were constructed, including fasting glucose (*fasting*) (which in the single-variable analyses proved to have greater predictive value than admission glucose) and other variables of known prognostic value: *age*, heart failure (*killip*), ST-segment elevation myocardial infarction (*stemi*), *sex*, *smoking*, previous coronary artery disease (*pcad*), coronary angiography (*cang*), *creatinine,* and *anemia*. The analyses were performed for the two groups of patients: those with a diagnosis of diabetes mellitus (*diabetes = 1*) and those without (*diabetes = 0*).

The *dfmacox* function may be computationally demanding, especially for large sample sizes. Because of this, first, we recommend fitting univariate additive Cox models to study the influence of each covariate separately. This procedure suggests the inclusion of three continuous predictors (*fasting*, *creatinine,* and *age*) with nonlinear effects. Then, we use function *dfmacox* to get the optimal degrees of freedom for the natural cubic spline fit of these three covariates:

R> df2<-dfmacox (time = "time", status
 
= "exitus", nl.predictor = c ("fasting",
 
"age", "creatinine"), other.predictors
 
= c ("cang", "sex", "smoking", "stemi",
 
"pcad", "killip", "anemia"), smoother
 
= "ns", method = "AIC", data = heart2)
R> df2$df
 
[1] 3 1 2
R> fit.mvcox.1<-coxph (Surv (time, exitus)
 
~ns (fasting, df = df2$df[1])
 
+ ns (age, df = df2$df[2])
 
+ ns (creatinine, df = df2$df[3])
 
+ cang + sex + smoking
 
+ stemi + pcad + killip + anemia,
 
data = heart2, x = TRUE)
R> hr2<-smoothHR (data = heart2,
 
 coxfit = fit.mvcox.1)
R> xx<-c (66, 110, 162, 258, 354, 450)
R> plot (hr2, predictor = "fasting",
 
prob = 0,conf.level = 0.95, ref.label
 
= "Ref.", xaxt = "n", main = "", xlab
 
= "Fasting glucose (mg/dL)")
R> axis(1, xx)

**Figure 2:**
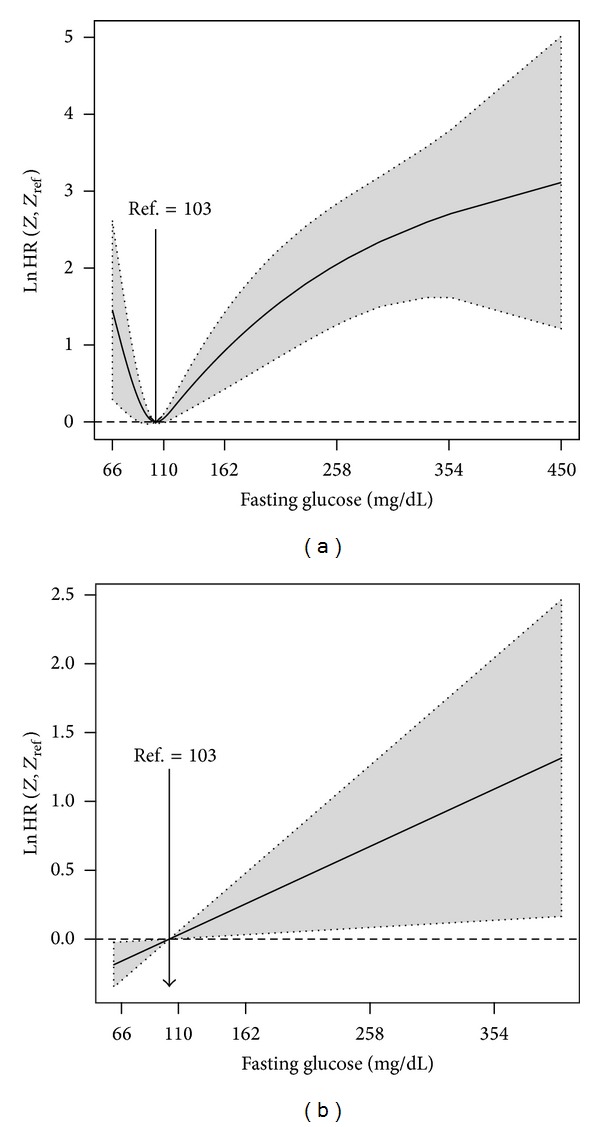
Nonparametric estimates of the dependence of all-time risk of death on fasting glucose among ACS patients with (b) and without (a) a prior diagnosis of diabetes mellitus (log hazard ratio, with 95% confidence limits).

Among those patients with no history of diabetes the J-shaped dependence of the all-time mortality hazard ratio on fasting glucose persisted when adjusted in a multivariable Cox model (see plot on the left hand side of Figure 2). A minimum risk is now achieved at 103 mg/dL.

However, fasting glucose levels did not reveal itself as a good predictor among patients with diabetes in regard to risk of death. This can be seen in the Cox model fitted below (*fit*.*mvcox*.2; results not shown). For comparison purposes we present in Figure 2 the log hazard ratio curve for those patients using the reference value of 103:

R> heart3<-heart [heart$diabetes==1,]
R> df3<-dfmacox (time = "time", status
 
= "exitus", nl.predictors = c
 
("fasting","creatinine", "age"),
 
 other.predictors = c ("cang", "smoking",
 
"stemi", "sex", "pcad", "killip",
 
"anemia"), smoother = "ns", method
 
= "AIC", data = heart3)
R> fit.mvcox.2<-coxph (Surv (time, exitus)
 
~ns (fasting, df3$df[1]) + ns
 
(creatinine, df3$df[2]) + ns
 
(age, df3$df[3]) + cang + smoking
 
+ stemi + sex + pcad + killip + anemia,
 
data = heart3, x = T)
R> hr3<-smoothHR (data = heart3, coxfit
 
= fit.mvcox.2)
R> xx<-c (66, 110, 162, 258, 354, 450)
R> plot (hr3, predictor = "fasting",
 
 pred.value = 103, conf.level = 0.95,
 
 ref.label = "Ref.", xaxt = "n", main
 
 = "", xlab = "Fasting glucose (mg/dL)")
R> axis(1, xx)

### 5.2. Breast Cancer Data

In the paper by Cadarso-Suárez et al. [9] the Galician breast cancer data is analyzed using a 3-state progressive model. In this section we will focus on various factors for predicting recurrence. Cadarso-Suárez et al. [9] found that DNA index (DI, the ratio of the G0/G1 channel number of tumor cells to the G0/G1 channel number of diploid cells), tumor size (size, measured in mm), and LNI (lymph node involvement) were important prognostic factors and that their effect should be introduced nonlinearly. Additional important prognostic factors were SPF (percentage of cells in phase S, in which the cell duplicates its DNA) and ER (hormone receptor status).

In order to implement the Cox model (3), first we need to find the degrees of freedom for the three continuous covariates to be introduced with a nonlinear (P-spline) effect. For this purpose, we use the *dfmacox* function to obtain the degrees of freedom minimizing the corrected AIC. Below, we present the corresponding input command. In addition, we show the degrees of freedom attained, the corresponding value (score) for the AIC, and the corresponding fitted model:

R> dfbreast<-dfmacox (time = "time_rec",
 
 status = "rec", nl.predictors = c ("DI",
 
"size", "LNI"), other.predictors = c
 
("SPF","ER"), smoother = "pspline",
 
 method = "AICc", data = breast)
R> dfbreast$df
 
[1] 14.987432 10.6316311.503889
R> dfbreast$AIC
 
[1] 1506.235
R> dfbreast$myfit

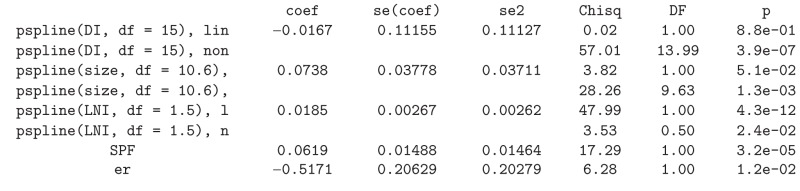


Iterations: 5 outer, 21 Newton-Raphson

Theta = 0.268

Theta = 0.0344

Theta = 0.938

Degrees of freedom for terms = 15.0 10.6

1.5 1.0 1.0

Likelihood ratio test = 228 on 29.1 df, p = 0

n = 498 (86 observations deleted due to

missingness)

Results are in good agreement with those obtained by Cadarso-Suárez et al. [9].

**Figure 3:**
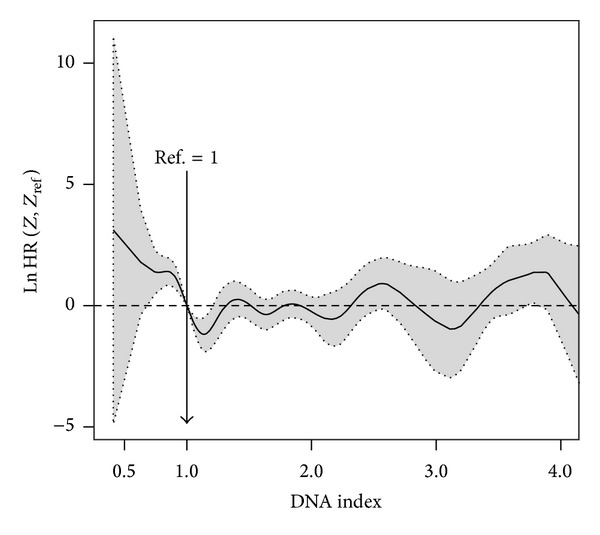
Nonparametric estimates of the dependence of all-time risk of recurrence on DNA index among patients with breast cancer (log hazard ratio, with 95% confidence limits). Reference value = 1.

**Figure 4:**
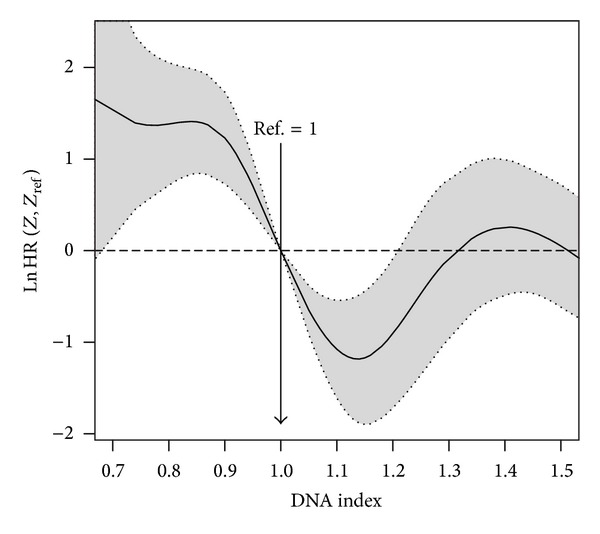
Nonparametric estimates of the dependence of all-time risk of recurrence on DNA index (restricted to the interval between 0.7 and 1.5) among patients with breast cancer (log hazard ratio, with 95% confidence limits). Reference value = 1.

Smooth log hazard ratio estimates with 95% pointwise confidence limits for DNA index (DI) can be obtained using a reference value of 1. The corresponding input commands are displayed below and the corresponding plot is shown in Figure 3. The corresponding log HR curve depicted in Figure 3 reveals that the risk of recurrence diminishes sharply until a value of 1.13, then increases until a value of 1.4, and then remains roughly constant. These features can be seen more clearly in Figure 4. In this plot we restrict the *x*-axis to the interval 0.7–1.5. Numerical results can also be obtained for this (and other) interval using the *predict* function (see the corresponding input commands below):

R> fit.mvcox.3<-coxph (Surv (time_rec,
 
rec) ~pspline (DI, dfbreast$df[1])
 
+ pspline (size, dfbreast$df[2])
 
+ pspline (LNI, dfbreast$df[3])
 
+ SPF + ER, data = breast, x = T)
R> hr4<-smoothHR (data = breast, coxfit
 
= fit.mvcox.3)
R> xx<-c (0.5, 1, 2, 3, 4)
R> plot (hr4, predictor = "DI", conf.level
 
= 0.95, pred.value = 1, xlim
 
= c (0.4, 4), ref.label = "Ref.", xaxt
 
= "n", main = "", xlab = "DNA Index")
R> axis (1, xx)
R> xx<-c (0.7, 0.8, 0.9, 1, 1.1, 1.2, 1.3,
 
1.4, 1.5)
R> plot (hr4, predictor = "DI",
 
conf.level = 0.95, pred.value = 1,
 
xlim = c (0.7,1.5), ref.label = "Ref.",
 
xaxt = "n", main = "",
  
xlab = "DNA Index")
R> axis(1, xx)
R> predict (hr4, predictor = "DI",
 
pred.value = 1, conf.level = 0.95,
 
prediction.values = xx,
 
ref.label = "Ref.")

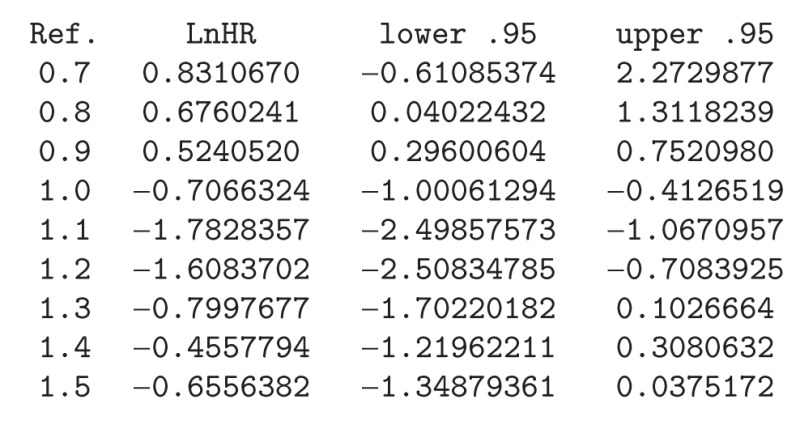


We should mention that direct comparisons with the results and plots provided by Cadarso-Suárez et al. [9] should be taken with care since the two fits are based on different sample sizes. This is due to the fact that the covariate SBR with missing data was excluded in the present study, so our fitted model is based on a larger sample (*n* = 498 against *n* = 421).

### 5.3. Comparison Study of the Choice of Degrees of Freedom

It is well known that, in general, the AIC has a big chance of choosing too much degrees whereas the BIC often chooses models with few degrees of freedom. The corrected AIC (AICc) can be considered as an alternative to both. In this section we compare the attained degrees of freedom from the three criteria (AIC, AICc, and BIC). For comparison purposes we also include the degrees of freedom obtained automatically using REML (using the software BayesX; Kneib and Fahrmeir [17], and Cadarso-Suárez et al. [9]). This procedure was considered using the two data sets with the same set of covariates for each multivariable Cox model (fit.mvcox.1 and fit.mvcox.3).

Table 2 shows the degrees of freedom obtained for the multivariable Cox model with penalized splines for fasting, creatinine and age (acute coronary syndrome data). As for model *fit*.*mvcox*.1, cang, sex, smoking, stemi, pcad, killip, and anemia were the remaining predictors. Results confirm that the AIC-based method leads to a large number of degrees of freedom when compared to AICc, BIC, and REML. On Table 3 we show the values (scores) obtained for criteria AIC, AICc, and BIC (rows) for each Cox model with degrees of freedom obtained from minimization of the corresponding criterion (see the degrees of freedom obtained in Table 2). From this table we can see that the *dfmacox* function is indeed obtaining an optimal model in the sense of minimizing the corresponding criterion. For all four models (AIC, AICc, BIC, and REML), the AIC score is lower for the model with degrees of freedom based on the AIC criterion (using function *dfmacox*). Similarly, the AICc score is lower for the AICc model and the BIC score is lower for the BIC model. REML is not minimizing none of the three criteria. Aside from the AIC criterion all other criteria lead to similar plots for the log HR curves and similar results in terms of the significant predictive capability (*P* values).

A similar study was performed for the breast cancer data. Tables 4 and 5 report the analogous results attained in Tables 2 and 3 for the acute coronary syndrome data. The degrees of freedom shown in Table 4 were obtained using the same set of covariates as in model *fit*.*mvcox*.3. Again, the AIC-based method leads to more degrees of freedom than BIC and REML which is reflected in the corresponding log HR curves. Results for the four multivariable Cox models with degrees of freedom based on the different criteria reveal that all models are very similar in terms of the significant predictive capability (*P* values). For this set of covariates the corrected AIC (AICc) still obtains higher values for the degrees of freedom of some covariates. The problem with the big number of degrees of freedom can be seen in the variability shown in the right tail of Figure 3. However, this can be controlled using the argument Boundary.knots in function *pspline*. However, it seems that this figure captures quite well the effect of DNA index (DI) around 1 (as shown in Figure 4). The results reported in Table 5 confirm that the *dfmacox* function is indeed obtaining an “optimal” model in the sense of minimizing the corresponding criterion.

For comparison purposes we also obtain the degrees of freedom and the AIC score using the *pspline* function with the argument df = 0 (i.e., supposed to use the AIC criterion). The fitted Cox model attained a AIC score of 1528.04 larger than the results attained with function *dfmacox*:

R> fit<-coxph (Surv (time_rec, rec)
 
~pspline (DI, df = 0) + pspline
 
(size, df = 0) + pspline (LNI, df = 0)
 
+ SPF + ER, data= breast, x = T)
R> fit$df
 
[1] 15.9151082 10.8817302
 
16.9549295 0.9984411 0.9999322
R> −2∗fit$loglik [2] + 2∗sum (fit$df)
 
[1]1528.04

## 6. Discussion

This paper gives an overview of the smoothHR package for the computation of pointwise estimates of HR curves as well as the corresponding confidence limits for continuous predictors introduced nonlinearly in an additive multivariable Cox regression model. This function provides both numerical and graphical output. In this paper, spline based approaches (natural cubic splines and P-splines) were used as the smoothing technique. Although these smoothing approaches have shown to be a good option, other smoothers can also be used in this context.

When using spline smoothing, special attention is called for when selecting the optimal amount of smoothing. The package includes an R-function that considers the methodology of Eilers and Marx [8] to implement AIC-based criterion in survival analysis. The software also enables the user to obtain degrees of freedom using the corrected AIC criterion proposed by Hurvich et al. [11] and the BIC criterion by Volinsky and Raftery [13]. An alternative approach that allows for determining smoothing parameters in the multivariable setting has been proposed by Kneib and Fahrmeir [17] based on a mixed model representation of penalized splines. It may be worthwhile to conduct a comparative study to compare the two approaches. We have also illustrated the proposed methods using real data. The analysis of the real data revealed that the minimization of these criteria, can lead to significant differences in the choice of the degrees of freedom. A compromise between the corrected AIC criterion, the BIC criterion and prior ideas of biologic plausibility is recommended.

An interesting open question is to generalize the application of these ideas to more complex additive Cox models with different smoothers or with a smooth baseline. The amount of smoothing can also be determined using cross-validation (CV) or generalized cross-validation (GCV). However, these may fail to work if the number of smoothing parameters is large as then the computational effort to compute an optimal solution becomes intractable. These are topics of current work and hopefully will be implemented in the future.

## Figures and Tables

**Table 1:** Summary of functions in the package.

Function	Description
smoothHR	Main function of the package. Returns an object of class HR.
dfmacox	Provides the number of degrees of freedom in the additive Cox model.
plot	A function that provides the plots for the hazard ratio curves taking a specific value as reference.
predict	Provides estimates for the hazard ratio and their corresponding confidence limits.
print	Prints details about the Cox model.

**Table 2:** Degrees of freedom (df) for the multivariable Cox model with penalized splines for fasting, creatinine, and age (cang, sex, smoking, stemi, pcad, killip, and anemia were the remaining predictors). Acute coronary syndrome data.

Covariates	dfAIC	dfAICc	dfBIC	dfREML
Fasting	4.80	3.62	1.59	2.57
Creatinine	7.97	1.48	1.49	2.04
Age	9.50	1.56	1.56	2.51

**Table 3:** Values obtained for criteria AIC, AICc, and BIC (rows) for the corresponding Cox models (columns). Acute coronary syndrome data.

	Model	AIC	AICc	BIC	REML
Score					
AIC		858.096	860.048	862.907	861.898
AICc		899.816	869.928	870.774	872.283
BIC		930.326	895.101	893.166	898.032

**Table 4:** Degrees of freedom (df) for the multivariable Cox model with penalized splines for DI, size, and LNI. SPF and ER were the remaining predictors. Breast cancer data.

Covariates	dfAIC	dfAICc	dfBIC	dfREML
DI	14.99	14.99	5.10	6.39
Size	10.98	10.63	1.78	2.78
LNI	2.01	1.50	1.50	2.18

**Table 5:** Values obtained for criteria AIC, AICc, and BIC (rows) for the corresponding Cox models (columns). Breast cancer data.

	Model	AIC	AICc	BIC	REML
Score					
AIC		1505.559	1506.235	1539.252	1537.230
AICc		1524.557	1524.200	1543.318	1542.556
BIC		1595.218	1593.327	1570.269	1577.272
